# Development and preliminary psychometric evaluation of the symptoms of autistic depression—parent scale

**DOI:** 10.3389/fped.2026.1735273

**Published:** 2026-04-17

**Authors:** Brianne Derby, Lindsey Wilson, Elizabeth Kelley

**Affiliations:** 1 Department of Psychology, Queen's University, Kingston, ON, Canada; 2 Department of Psychiatry, Queen's University, Kingston, ON Canada; 3 Providence Care Hospital, Kingston, ON Canada; 4 Departments of Psychology and Psychiatry, Queen's University, Kingston, ON, Canada

**Keywords:** autism, comorbidity, depression, depression measures, factor analysis

## Abstract

**Introduction:**

The current study preliminarily assessed the psychometric properties of the Symptoms of Autistic Depression—Parent (SAD-P) scale.

**Method:**

The SAD-P and the Child Behaviour Checklist (CBCL) were administered to 196 parents of autistic and neurotypical youth.

**Results:**

A preliminary exploratory factor analysis (EFA) suggested a 1-factor model best suited the SAD-P based on factor loadings, parsimony, and interpretability. EFAs conducted separately for parents of neurotypical youth and autistic youth also supported a one-factor solution. The SAD-P demonstrated acceptable convergent validity (*r* =.65) with the CBCL Depression subscale and good discriminant validity (*r* =.42) with the Conduct Problems subscale.

**Discussion:**

Although this study is a preliminary step in scale development and more research is needed to validate the scale, the SAD-P shows promise as a preliminary measure of depressive symptoms in both autistic and neurotypical youth.

## Introduction

Co-occurring symptoms in autistic populations can present differently than in neurotypical populations, making them difficult to assess and treat (1). Prevalence rates of depression in autistic communities vary widely. Meta-analytic findings show that depressive disorders are more common among individuals with autism. However, prevalence estimates vary widely across studies, reflecting variation in sampling strategies (e.g., population-based vs. clinically referred samples), age composition, assessment time frame (current vs. lifetime diagnoses), and methods used to identify depression. Hudson et al. (2) synthesized prevalence rates across methodologically diverse studies, emphasizing that broad ranges cannot be meaningfully compared without accounting for these moderating factors. More recently, Mutluer et al. (3) provided population-based estimates in pediatric samples and identified higher rates of depression during adolescence, offering a clearer epidemiological reference point for measurement research in youth. Together, these studies underscore the need for autism-sensitive assessment tools. Some research has found that depression is four times more prevalent in autistic than neurotypical youth when interviews were used (4). Prevalence rates tend to vary significantly based on the type of assessment used (2). Assessing depression is difficult due to poor diagnostic classification and the overlap in core autistic traits and common symptoms of depression (5). This overlap can cause diagnostic overshadowing, in which clinicians falsely dismiss symptoms of a co-occurring disorder (i.e., symptoms of depression) as symptoms of the individual's primary disorder (i.e., autism) (6).

Some depressive symptoms may also present differently in autistic populations. Symptoms of irritability, compulsive behaviour, agitation, and aggression have been found to be more commonly- endorsed depressive symptoms in autistic individuals (7). Despite some differences in depressive symptom presentation between autistic and neurotypical populations, some symptoms of depression in autistic individuals are similar to symptoms commonly seen in the neurotypical population (8). Despite differences in the presentation of depression in autism, only two studies, to our knowledge, have created a preliminary measure to assess depressive symptoms as seen in autistic people. However, one scale was only created in French with a small sample size and no examination of convergent and divergent validity (9), while the other was for autistic adults (10).

Measures designed for the general population may not be accurately assessing co-occurring depression in autism. Considering depression in autistic youth can increase the risk of self-harm and suicidal ideation it is imperative to create an assessment of depressive symptoms specifically for autistic youth (6, 8).

## Current study

We designed a new preliminary measure for autistic youth with the aim of addressing the concerns surrounding the current measures of depressive symptoms. Questions in the Symptoms of Autistic Depression-Parent (SAD-P) measure were created based on literature describing autistic presentations of depression (2, 5, 11) and the DSM-5 criteria of depression. We also examined commonly used depression measures for youth. The last author, who has been working in the field of autism for 35 years oversaw the development of this measure. See Table 1 for the origin of each item.

To address the concern of diagnostic overshadowing and to account for the overlap in symptoms of depression and autism, symptoms should be compared to baseline behaviours (12). As such, this new preliminary measure asked parents questions about the adolescent's symptoms within the past two weeks compared to what is typical for them. To address signs of depression in those with high support needs and/or severe or profound intellectual disability, questions were asked about a regression in previously-attained skills (5).

In this study, we gave the new SAD questionnaire to parents of youth ages 12–18 years old, alongside the Child Behaviour Checklist (CBCL) (13). Our first objective was to identify the factor structure of the SAD-P and remove any poorly performing items. Our next objective was to examine the validity of and the internal consistency of the SAD-P. We hypothesized that the SAD-P would be correlated over .70 with the Depression subscale, suggesting convergent validity. However, because the Conduct Problems subscale in the CBCL measures rule-breaking and aggressive behaviours, we predicted the relationship between the SAD-P and the Conduct Problems subscale would be correlated below .50, suggesting discriminant validity (14). We hypothesized that the SAD-P would demonstrate adequate internal consistency (i.e., Omega > .70).

## Materials and methods

### Participants

Our study recruited parents of youth ages 12–18 years old. The first group consisted of 72 parents of neurotypical youth, and the second group consisted of 124 parents of autistic youth. No restrictions were in place regarding ethnicity, gender identity, IQ, or level of support needs, and participants' children were not restricted by the presence of co-occurring conditions. Neurotypical participants' children could not have a neurodevelopmental condition but could have a psychiatric condition. Participants were recruited from across Canada and the United States through Facebook ads, having service providers and researchers send out flyers to families, and from our own databases of families that had previously participated in research. More demographic information can be found in Table 2. Our study received ethics approval from the General Research Ethics Board (GREB)in accordance with the 1964 Declaration of Helsinki and all amendments thereof. All parents provided informed consent.

**Table 1:** Origins of items for questionnaire.

1. How often did your child have a good time? CDI
2. How often did your child look upset? DSM-5
3. How often did your child appear to think that things often happened the way they wanted them to? CDI
4. How often did your child have problems with their friends? CDI
5. How often did your child not enjoy anything? DSM-5
6. How often did your child express that they felt happy about the future? DSM-5
7. How often did your child act restless? DSM-5
8. How often was your child grumpy? DSM-5
9. In the past two weeks, how often did your child express that they felt like they were just no good anymore? CDI
10. How often did your child express that they feel loved by their family? CDI
11. How often did your child want to see their friends? CDI
12. How often was your child happy with the person they are? CDI
13. How often was your child easily irritated or bothered? DSM-5
14. How often did your child express that they felt like they physically couldn't do things that they used to be able to do? CDI
15. How often was your child able to control their emotions easily? Magnusson & Constantino, 2011
16. How often was your child moving slower than usual? DSM-5
17. How often did your child express that they feel like they have no one? CDI
18. How often did your child express that they feel like a failure? CDI
19. In the past two weeks, how often did your child have a hard time concentrating on tasks such as schoolwork or chores? DSM-5
20. How often was your child happy one minute and sad the next? Magnuson & Constantino, 2011
21. How often was your child sleeping more or less than usual? DSM-5
22. How often did your child express that they feel that their family would be better off without them? Feelings expressed by autistic teen EK worked with
23. How often was your child hyper? DSM-5
24. How often did your child dislike the way they look? CDI
25. How often did your child look happy? DSM-5
26. How often did your child feel guilty for something that wasn't their fault? DSM-5
27. How often did your child talk less than usual? DSM-5
28. How often did your child have a hard time making decisions? DSM-5
29. In the past two weeks, how often did your child express that they thought bad things would happen? CDI
30. How often did your child enjoy being around others? CDI
31. How often did your child express that they hated themselves? CDI
32. How often did your child cry a lot? DSM-5
33. How often was your child motivated to complete tasks that they wanted to do? CDI
34. How often did your child express that they feel like their family does not care about them? CDI
35. How often did your child seem tired at an unusual time? CDI
36. How often did your child express that they had a hard time coming up with happy thoughts? Magnusson & Constantino, 2011
37. How often was your child eating more or less than usual? DSM-5
38. How often did your child lose interest in things that previously brought them a lot of happiness? DSM-5
39. In the past two weeks, how often was your child happy with who they are? DSM-5
40. How often did your child feel lonely? CDI
41. How often did your child get easily frustrated? DSM-5
42. How often did it appear that your child did not want to move around? DSM-5
43. How often did your child not enjoy things as much as they used to? DSM-5
44. How often did your child express that they feel worthless? DSM-5
45. How often was your child physically or verbally aggressive to someone else? Stewart et al, 2006
46. How often could your child make decisions easily? DSM-5
47. How often did your child blame themselves for things that weren't their fault? CDI
48. How often did your child appear to have a hard time thinking properly? DSM-5
49. In the past two weeks, how often did your child appear to have sad thoughts over and over again? DSM-5
50. How often did your child have tummy aches, and/or headaches, and/or body pain? CDI
51. How often did your child feel like good things would happen? CDI
52. How often is your child wetting the bed at night? Stewart et al., 2006
53. How often if your child unable to dress themselves? Stewart et al., 2006
54. How often does your child struggle to maintain personal hygiene without prompting? Stewart et al., 2006
55. How often did you think that your child's ADHD/ASD symptoms were getting worse? Stewart et al, 2006

DSM-5, diagnostic and statistical manual of psychiatric disorders; CDI, childhood depression inventory.

**Table 2:** Demographic information for parent and youth samples.

Sample Characteristics	Total parent sample				ASD parent sample				NT parent sample			
	*n*	*%*	*M*	*SD*	*n*	*%*	*M*	*SD*	*n*	*%*	*M*	*SD*
Sample size	196				124				72			
Age of youth			14.47	1.83			14.61	1.85			14.22	1.78
Gender of youth												
Male	111	57			82	66			29	40		
Female	83	42			40	32			43	60		
Non-binary	2	1			2	2			0	0		
SAD-P Depression Score*			1.50	0.18			1.53	0.19			1.47	0.15
Diagnosis of youth												
Neurotypical	72	37										
ASD	124	63										
Youth with Co-occurring ASD and ADHD	60	48			60	48			0	0		
Diagnosis of Psychiatric Disorder(s) in Youth	54	27			51	41			3	4		
Anxiety	43	22			40	32			3	4		
Depression	13	7			13	10			0	0		
Conduct Disorder	1	.5			1	1			0	0		
Bipolar Disorder	3	2			3	2			0	0		
Tourette's	1	.5			1	1			0	0		
OCD	9	5			9	7			0	0		
Learning Disability	1	.5			1	1			0	0		
Dysthymia	1	.5			1	1			0	0		
Multiple Disorders	18	9			18	15			0	0		
Relationship to Youth												
Mother	169	86			104	84			65	90		
Father	19	10			12	10			7	10		
Other**	8	4			8	6			0	0		
Parental education												
High School	2	1			1	1			1	1		
Some College	8	4			4	3			4	6		
College	40	20			27	22			13	18		
Some University	13	7			9	7			4	5		
University	69	35			42	34			27	38		
Post-Graduate Degree	64	33			41	33			23	32		

Parents responded to demographic questions related to their child's age, diagnosis, and presence of any psychiatric disorder, along with questions about their relationship to the child and their (parental) education.

* Total SAD-P depression scores across all parent reports ranged from 1.19 to 2.13. Within this overall range, scores for the ASD group ranged from 1.19 to 2.13, whereas scores for the neurotypical (NT) group ranged from 1.25 to 1.93.

** Other relationships to youth included stepparents, both mother and father, and grandparents.

### Measures and procedure

Participants met with a research assistant over Zoom. Parents began by responding to the SAD-P, followed by the CBCL. In the SAD-P, parents answered questions related to their child's mood over the past two weeks. The SAD-P had two types of questions: positive and negative questions. Positive questions were related to positive experiences or emotions. Negative questions were related to negative experiences or emotions. The parents had four responses: *Never* (1), *Sometimes* (2), *Often* (3), or *Almost Always* (4). One question (“How often did you think that your child's ASD symptoms were getting worse?”) was given to only parents of autistic youth, resulting in 55 questions for parents of autistic youth and 54 questions for parents of neurotypical youth. Positive questions (13 items) were reverse coded so that higher scores indicated higher levels of depression (*Never* = 4, *Sometimes* = 3, *Often* = 2, *Almost Always* = 1). Total depression scores were computed as the mean of all item responses for each participant. Items deemed not applicable or skipped by the respondent were excluded from the calculation in the total score. Higher scores indicated higher depressive symptoms, with a lowest possible total score of 1.0 and a highest possible score of 4.0 (see Total SAD-P Depression scores in Table 1). The CBCL/6-18 has been validated in autistic samples and has shown to be a reliable measure of behavioural and emotional difficulties in autistic children and youth (15). Parents responded to 112 questions from the “Problem Items” section in the CBCL for ages 6 to 18 (13).

### Statistical analysis

We preformed exploratory factor analyses (EFAs) to examine the factor structure of the SAD-P. We conducted a principal axis factor analysis of the SAD-P using the psych package in R (16). Missing data were handled using Full Information Maximum Likelihood (FIML) method. To determine the number of factors to extract, we used a multiple model approach relying on several different criteria to identify the best model (17). We examined the scree plot and conducted a parallel analysis. General recommendations for evaluating model fit are that the Root Mean-Square Error of Approximation (RMSEA) should be less than or equal to .05 for good fit or less than .10 for acceptable fit, the Root Mean Square of Residuals (RMSR) should be less .08, and the Tucker Lewis Index (TLI) should be greater than .95 for good fit (18). However, these guidelines were based on a three-factor CFA model and have been overgeneralized to other models, like EFA, to which they were not intended to apply (19). Montoya and Edwards (20) concluded that fit indices should not be used to determine the number of factors in EFA, and Finch (2020) (21) recommended using changes in RMSEA of 0.015 (but not overall levels or changes in the other fit indices) for factor extraction in EFA. To gain a comprehensive approach to determining the factor structure of the SAD, we examined RMSEA, RMSR, and TLI fit indices^1^, the parsimony and interpretability of each factor analysis and changes in RMSEA.

After removing the items that did not load substantially in the EFA for the parent sample, we examined the reliability of the SAD-P using Omega. An omega of .70 or higher was considered acceptable reliability. Finally, we examined the convergent and discriminant validity of the SAD-P by correlating total depression scores in the SAD-P to Depression T-scores and Conduct Problems T-scores in the CBCL. We used the Fisher's Z-transformation test to determine if the correlations were significantly different from one another.

## Results

The scree plot suggested five factor models (1-, 2-, 3-, 4-, and 5-factors) warranted further examination, whereas the parallel analysis suggested a seven-factor model would be the best. As such, we tested six different factor solutions (1-, 2-, 3-, 4-, 5-, and 7-factors) using polychoric correlations and an oblique (oblimin) rotation. All the EFA models had acceptable fit based on the RMSEA but not good fit until seven factors were retained, and only the one-and two-factor models were above the cut-off for the RMSR. None of the models had good fit according to the TLI (Table 3). The one-factor model had the fewest insubstantially loading items and cross loadings. While the higher factor models demonstrated better fit statistics than the one-factor model, many items within these models loaded insubstantially or loaded highly onto multiple factors, and the factors were not interpretable as a construct. This is an indication of over-factoring and is consistent with Montoya and Edwards' (20) findings that fit indices favour more factors and thus relying on model fit in EFA leads to over-factoring. Based on interpretability and parsimony, the one-factor model appeared to be the best model for the SAD-P (Table 4). Any items that did not load above |.3| on the one factor were removed from the questionnaire in subsequent analyses (see Tables 5, 6 for the 5- and 7-factor models).

**Table 3:** Goodness of Fit indices for the 1-, 2-, 3-, 4-, 5- and 7-factor models using All parent data.

Model structure	RMSEA	RMSR	TLI
1-Factor Model	.09	.09	.56
2-Factor Model	.08	.08	.61
3-Factor Model	.07	.07	.68
4-Factor Model	.06	.06	.74
5-Factor Model	.06	.05	.77
7-Factor Model	.05	.04	.82

**Table 4:** Factor loadings from the EFA using all parent data: 1-factor model.

Item	Factor 1	*h* ^2^
Have a good time?	**–0**.**54**	.29
Look upset?	.**69**	.48
Think things happened the way they wanted them to?	**–0**.**61**	.37
Have problems with their friends?	.**48**	.23
Not enjoy anything?	.**57**	.32
Feel happy about the future?	**–0**.**42**	.17
Act restless?	.**38**	.15
Grumpy?	.**61**	.37
Feel like they were just no good anymore?	.**72**	.52
Feel loved by their family?	**–0**.**31**	.09
Happy with the person they are?	**–0**.**77**	.60
Easily irritated or bothered?	.**63**	.40
Felt like they physically couldn't do things that they used to be able to do?	.**46**	.21
Able to control their emotions easily?	**–0**.**56**	.31
Feel like they have no one?	.**61**	.37
Feel like a failure?	.**75**	.57
Have a hard time concentrating on tasks such as schoolwork or chores?	.**50**	.25
Happy one minute and sad the next?	.**61**	.37
Sleeping more or less than usual?	.**41**	.17
Feel that their family would be better off without them?	.**61**	.37
Dislike the way they look?	.**41**	.17
Look happy?	**–0**.**66**	.43
Feel guilty for something that wasn't their fault?	.**34**	.12
Talk less than usual?	.**40**	.16
Have a hard time making decisions?	.**40**	.16
Express that they thought bad things would happen?	.**61**	.37
Enjoy being around others?	**–0**.**39**	.15
Express that they hated themselves?	.**67**	.44
Cry a lot?	.**49**	.24
Motivated to complete tasks that they wanted to do?	**–0**.**39**	.15
Feel like their family does not care about them?	.**56**	.32
Seem tired at an unusual time?	.**40**	.16
Have a hard time coming up with happy thoughts?	.**69**	.48
Eating more or less than usual?	.**33**	.11
Happy with who they are?	**–0**.**77**	.59
Feel lonely?	.**62**	.38
Easily frustrated?	.**62**	.38
Not want to move around?	.**40**	.16
Not enjoy things as much as they used to?	.**61**	.37
Feel worthless?	.**69**	.47
Physically or verbally aggressive to someone else?	.**43**	.18
Make decisions easily?	**–0**.**43**	.18
Blame themselves for things that weren't their fault?	.**45**	.21
Have a hard time thinking properly?	.**50**	.25
Have sad thoughts over and over again?	.**70**	.49
Have tummy aches, and/or headaches, and/or body pain?	.**46**	.22
Feel like good things would happen?	**–0**.**65**	.42
Want to see their friends?	**–**0.19	.04
Moving slower than usual?	.28	.08
Hyper?	.08	.01
Lose interest in things that previously brought them a lot of happiness?	.24	.06
Wetting the bed at night?	.01	.00
Unable to dress themselves?	**–**0.08	.01
Struggle to maintain personal hygiene without prompting?	.27	.07

All questions began with “How often did/was your child…”. *h*^2^ = commonality estimated with the psych package in R from the unrotated solution. Bolded loadings identify substantial loadings. Items with no bolded loadings had no loading greater than |.30| and were removed from subsequent analyses.

**Table 5:** Factor loadings from the EFA using all parent data: 5-factor model.

Item	Factor 1	Factor 2	Factor 3	Factor 4	Factor 5	*h* ^2^
Have a good time?	.10	.**70**	–0.08	–0.05	–0.05	.52
Look upset?	.**37**	–0.20	.38	–0.01	.00	.51
Think things happened the way they wanted them to?	–0.16	.**34**	–0.13	–0.14	–0.11	.37
Have problems with their friends?	.19	.02	.**30**	.12	.17	.28
Not enjoy anything?	.02	–0.**43**	.20	.21	–0.06	.40
Feel happy about the future?	.02	.**70**	.10	.05	.01	.43
Act restless?	–0.09	.05	.**51**	.15	.12	.32
Grumpy?	.20	–0.**30**	.**47**	–0.07	–0.03	.50
Feel like they were just no good anymore?	.**65**	–0.21	.11	.00	–0.11	.62
Feel loved by their family?	–0.01	.**64**	.03	.07	.16	.37
Happy with the person they are?	–0.**32**	.**53**	–0.02	–0.09	–0.11	.66
Easily irritated or bothered?	.04	–0.22	.**57**	–0.03	.15	.53
Felt like they physically couldn't do things that they used to be able to do?	.**30**	.04	–0.01	.**30**	.05	.24
Able to control their emotions easily?	–0.08	.11	–0.**58**	–0.03	–0.17	.52
Feel like they have no one?	.**52**	–0.12	.07	.10	.00	.43
Feel like a failure?	.**71**	–0.05	.04	.14	.00	.66
Have a hard time concentrating on tasks such as schoolwork or chores?	–0.04	.00	.**39**	.41	.02	.39
Happy one minute and sad the next?	.**35**	–0.01	.**34**	.13	.05	.41
Sleeping more or less than usual?	–0.11	–0.20	–0.14	.**58**	.06	.38
Feel that their family would be better off without them?	.**71**	.03	.03	.05	–0.02	.53
Dislike the way they look?	.**41**	–0.11	–0.21	.04	.26	.32
Look happy?	–0.01	.**78**	–0.05	–0.10	.04	.69
Feel guilty for something that wasn't their fault?	.**42**	.08	–0.25	.01	.**41**	.39
Talk less than usual?	.08	–0.08	–0.09	.**40**	.06	.23
Have a hard time making decisions?	–0.02	.01	.16	.05	.**73**	.61
Express that they thought bad things would happen?	.**40**	–0.07	.16	.10	.07	.35
Enjoy being around others?	.09	.**54**	.11	–0.13	–0.08	.31
Express that they hated themselves?	.**78**	.02	.09	.01	–0.07	.62
Cry a lot?	.**35**	.20	.29	.12	.10	.29
Motivated to complete tasks that they wanted to do?	.03	.**30**	–0.01	–0.20	–0.12	.20
Feel like their family does not care about them?	.**60**	–0.03	.14	–0.09	–0.02	.40
Seem tired at an unusual time?	.10	.03	–0.06	.**59**	–0.07	.35
Have a hard time coming up with happy thoughts?	.**41**	–0.15	.07	.27	.03	.48
Eating more or less than usual?	–0.01	.11	–0.06	.**47**	.20	.28
Happy with who they are?	–0.**31**	.**60**	.02	–0.04	–0.16	.72
Feel lonely?	.**34**	–0.07	.07	.**35**	–0.06	.38
Easily frustrated?	.16	.07	.**65**	.04	.23	.61
Not want to move around?	.11	–0.09	.09	.**36**	–0.10	.23
Not enjoy things as much as they used to?	.21	–0.14	.08	.**42**	–0.03	.40
Feel worthless?	.**74**	–0.10	.04	.01	–0.08	.63
Physically or verbally aggressive to someone else?	.10	–0.02	.**61**	.05	–0.08	.44
Make decisions easily?	.17	.17	–0.16	–0.05	–0.**72**	.64
Blame themselves for things that weren't their fault?	.**60**	.04	–0.28	.03	.**38**	.56
Have a hard time thinking properly?	–0.01	.05	.**32**	.**49**	.03	.41
Have sad thoughts over and over again?	.**50**	–0.18	.13	.08	.03	.48
Have tummy aches, and/or headaches, and/or body pain?	.12	–0.03	.02	.**35**	.10	.23
Feel like good things would happen?	–0.06	.**77**	–0.05	.04	–0.08	.68

All questions began with “How often did/was your child…”. *h*^2^ = commonality estimated with the psych package in R from the unrotated solution. Bolded loadings identify substantial loadings. Items with no bolded loadings had no loading greater than |.30|. The seven items with unsubstantial loadings (below |.30|) in the 1-factor model were removed for this analysis.

**Table 6:** Factor loadings from the EFA using all parent data: 7-factor model.

Item	Factor 1	Factor 2	Factor 3	Factor 4	Factor 5	Factor 6	Factor 7	*h* ^2^
Have a good time?	.13	.**68**	–0.15	–0.03	–0.05	–0.04	–0.03	.53
Look upset?	.26	–0.17	.**48**	.00	.03	–0.06	.10	.54
Think things happened the way they wanted them to?	–0.16	.**32**	–0.05	–0.23	–0.04	–0.22	.11	.42
Have problems with their friends?	.05	.04	.28	.**30**	.00	.21	–0.03	.32
Not enjoy anything?	–0.04	–0.**42**	.27	.08	.21	–0.08	.01	.42
Feel happy about the future?	–0.10	.**68**	.09	.18	–0.03	–0.01	.00	.46
Act restless?	.11	.10	.**38**	–0.16	.22	.23	–0.19	.38
Grumpy?	.07	–0.27	.**62**	–0.04	–0.01	–0.11	.08	.57
Feel they were just not good anymore?	.**62**	–0.20	.11	.07	–0.01	–0.08	.02	.63
Feel loved by their family?	–0.14	.**61**	.09	.00	.08	.05	.20	.40
Happy with the person they are?	–0.15	.**52**	–0.12	–0.20	–0.02	–0.07	–0.15	.67
Easily irritated or bothered?	–0.02	–0.18	.**64**	–0.08	.04	.11	.02	.56
Feel like they physically couldn’t do things they used to be able to do?	.18	.04	–0.10	.**47**	.10	.15	–0.06	.35
Able to control their emotions easily?	–0.04	.07	–0.**53**	–0.09	–0.01	–0.23	.10	.52
Feel like they have no one?	.**44**	–0.11	–0.02	.**38**	–0.07	.11	–0.09	.53
Feel like a failure?	.**66**	–0.04	.05	.08	.11	.00	.13	.68
Have a hard time concentrating on tasks such as schoolwork or chores?	.19	.04	.28	–0.20	.**53**	.10	–0.14	.55
Happy one minute and sad the next?	.11	.00	.**40**	.**37**	.00	.05	.03	.47
Sleeping more or less than usual?	–0.08	–0.22	–0.12	.01	.**60**	.02	.14	.44
Feel that their family would be better off without them?	.**74**	.04	–0.02	.03	.04	.02	.05	.58
Look happy?	.02	.**75**	–0.09	–0.12	–0.07	.01	.04	.69
Feel guilty for something that wasn’t their fault?	.13	.06	.01	–0.12	.07	.13	.**72**	.62
Talk less than usual?	.02	–0.10	–0.02	.04	.**41**	–0.01	.19	.26
Have a hard time making decisions?	–0.05	.03	.08	.07	–0.01	.**74**	.13	.63
Enjoy being around others?	.04	.**53**	.09	.05	–0.15	–0.09	–0.04	.32
Express that they hated themselves?	.**78**	.04	.06	.04	.00	–0.04	.05	.66
Cry a lot?	.12	.19	.**35**	.26	.03	.06	.12	.32
Feel like their family does not care about them?	.**60**	–0.01	.13	–0.04	–0.06	–0.01	.06	.43
Seem tired at an unusual time?	–0.10	–0.01	.05	.26	.**53**	–0.17	.20	.42
Eating more or less than usual?	.09	.10	–0.16	.04	.**44**	.25	.03	.31
Happy with who they are?	–0.07	.**60**	–0.12	–0.24	.04	–0.08	–0.22	.76
Feel lonely?	.22	–0.08	.00	.**51**	.15	.04	–0.11	.50
Get easily frustrated?	.06	.10	.**65**	.08	.04	.22	.00	.62
Not want to move around?	.11	–0.09	.07	.16	.**30**	–0.05	–0.05	.22
Not enjoy things as much as they used to?	.09	–0.15	.06	.**40**	.27	.03	–0.04	.44
Feel worthless?	.**82**	–0.08	–0.02	–0.03	.02	–0.03	.02	.71
Physically or verbally aggressive to someone else?	.13	.02	.**56**	.02	.07	.00	–0.19	.43
Make decisions easily?	.09	.13	–0.03	.03	–0.02	–0.**81**	–0.04	.71
Blame themselves for things that weren’t their fault?	.**32**	.02	–0.09	.06	.02	.17	.**56**	.60
Have a hard time thinking properly?	.14	.07	.15	.11	.**44**	.18	–0.21	.44
Feel like good things would happen?	–0.16	.**73**	–0.04	.08	.01	–0.14	.05	.70
Dislike the way they look?	.22	–0.12	–0.14	.20	–0.05	.19	.26	.33
Think bad things would happen?	.22	–0.07	.28	.10	.09	–0.03	.22	.38
Motivated to complete tasks that they wanted to do?	–0.14	.28	.12	.03	–0.19	–0.23	.12	.25
Have a hard time coming up with happy thoughts?	.20	–0.16	.18	.25	.19	–0.04	.19	.50
Have sad thoughts over and over again?	.24	–0.18	.26	.25	.01	–0.05	.20	.51
Have tummy aches, and/or headaches, and/or body pain?	.04	–0.04	.01	.24	.25	.12	.06	.23

All questions began with “How often did/was your child…”. *h*^2^ = commonality estimated with the psych package in R from the unrotated solution. Bolded loadings identify substantial loadings. Items with no bolded loadings had no loading greater than |.30|. The seven items with unsubstantial loadings (below |.30|) in the 1-factor model were removed for this analysis.

We then compared the one-factor EFA solution for just the neurotypical parent reports vs. just the autistic parent reports. For the parents of neurotypical youth (*n* = 72), two items did not load above a |.30| (see Table 7), suggesting these items may not perform as well in assessing depression in neurotypical youth. The one-factor model for parent reports of neurotypical youth was consistent with the one-factor solution from the full parent sample.

**Table 7:** Factor loadings from the EFA using neurotypical parent data: 1-factor model.

Item	Factor 1	*h* ^2^
Have a good time?	**–0**.**53**	.28
Look upset?	.**67**	.44
Think things happened the way they wanted them to?	**–0**.**58**	.34
Have problems with their friends?	.**48**	.29
Not enjoy anything?	.**41**	.17
Feel happy about the future?	**–0**.**46**	.21
Grumpy?	.**52**	.27
Feel like they were just no good anymore?	.**65**	.43
Feel loved by their family?	**–0.35**	.13
Happy with the person they are?	**–0**.**68**	.46
Easily irritated or bothered?	.**62**	.39
Felt like they physically couldn't do things that they used to be able to do?	.**35**	.12
Able to control their emotions easily?	**–0**.**46**	.21
Feel like they have no one?	.**51**	.26
Feel like a failure?	.**65**	.42
Have a hard time concentrating on tasks such as schoolwork or chores?	.**53**	.28
Happy one minute and sad the next?	.**57**	.33
Sleeping more or less than usual?	.**31**	.10
Feel that their family would be better off without them?	.**46**	.21
Dislike the way they look?	.**61**	.37
Look happy?	**–0**.**67**	.44
Feel guilty for something that wasn't their fault?	.**43**	.19
Talk less than usual?	.**57**	.33
Have a hard time making decisions?	.**33**	.11
Express that they thought bad things would happen?	.**71**	.50
Enjoy being around others?	**–0**.**50**	.25
Express that they hated themselves?	.**64**	.41
Cry a lot?	.**48**	.23
Motivated to complete tasks that they wanted to do?	**–0**.**65**	.42
Feel like their family does not care about them?	.**52**	.27
Seem tired at an unusual time?	.**40**	.16
Have a hard time coming up with happy thoughts?	.**81**	.65
Eating more or less than usual?	.**32**	.11
Happy with who they are?	**–0**.**81**	.66
Feel lonely?	.**70**	.49
Easily frustrated?	.**57**	.33
Not want to move around?	.**32**	.11
Not enjoy things as much as they used to?	.**76**	.57
Feel worthless?	.**72**	.52
Make decisions easily?	**–0**.**34**	.12
Blame themselves for things that weren't their fault?	.**50**	.25
Have a hard time thinking properly?	.**53**	.28
Have sad thoughts over and over again?	.**76**	.58
Have tummy aches, and/or headaches, and/or body pain?	.**55**	.30
Feel like good things would happen?	**–0**.**70**	.50
Act restless?	.28	.08
Physically or verbally aggressive to someone else?	.16	.03

All questions began with “How often did/was your child…”. *h*^2^ = commonality estimated with the psych package in R from the unrotated solution. Bolded loadings identify substantial loadings. Items with no bolded loadings had no loading greater than |.30|.

Next, we examined the one-factor model in parents of autistic youth (*n* = 122). Four items did not load above a |.30| in this sample (see Table 8). The one-factor model for parents of autistic youth was consistent with the one-factor solution from the full sample.

**Table 8:** Factor loadings from the EFA using autistic parent data: 1-factor model.

Item	Factor 1	*h* ^2^
Have a good time?	**–0**.**50**	.25
Look upset?	.**68**	.46
Think things happened the way they wanted them to?	**–0**.**59**	.35
Have problems with their friends?	.**43**	.19
Not enjoy anything?	.**59**	.35
Feel happy about the future?	**–0**.**31**	.10
Act restless?	.**31**	.10
Grumpy?	.**61**	.38
Feel like they were just no good anymore?	.**73**	.53
Happy with the person they are?	**–0**.**81**	.66
Easily irritated or bothered?	.**55**	.30
Felt like they physically couldn't do things that they used to be able to do?	.**50**	.25
Able to control their emotions easily?	**–0**.**50**	.25
Feel like they have no one?	.**67**	.45
Feel like a failure?	.**78**	0.60
Have a hard time concentrating on tasks such as schoolwork or chores?	.**38**	.15
Happy one minute and sad the next?	.**61**	.37
Sleeping more or less than usual?	.**41**	.17
Feel that their family would be better off without them?	.**67**	.45
Dislike the way they look?	.**36**	.13
Look happy?	**–0**.**65**	.42
Talk less than usual?	.**33**	.11
Have a hard time making decisions?	.**32**	.10
Express that they thought bad things would happen?	.**54**	.30
Enjoy being around others?	**–0**.**30**	.09
Express that they hated themselves?	.**67**	.49
Cry a lot?	.**44**	.20
Feel like their family does not care about them?	.**59**	.35
Seem tired at an unusual time?	.**37**	.14
Have a hard time coming up with happy thoughts?	.**66**	.43
Happy with who they are?	**–0**.**74**	.55
Feel lonely?	.**59**	.35
Easily frustrated?	.**56**	.31
Not want to move around?	.**36**	.13
Not enjoy things as much as they used to?	.**55**	.30
Feel worthless?	.**68**	.47
Physically or verbally aggressive to someone else?	.**46**	.21
Make decisions easily?	**–0**.**38**	.15
Blame themselves for things that weren't their fault?	.**43**	.19
Have a hard time thinking properly?	.**38**	.14
Have sad thoughts over and over again?	.**68**	.46
Have tummy aches, and/or headaches, and/or body pain?	.**44**	.20
Feel like good things would happen?	**–0**.**57**	.32
Feel loved by their family?	–0.29	.09
Feel guilty for something that wasn't their fault?	.29	.08
Motivated to complete tasks that they wanted to do?	–0.25	.06
Eating more or less than usual?	.28	.08

All questions began with “How often did/was your child…”. *h*^2^ = commonality estimated with the psych package in R from the unrotated solution. Bolded loadings identify substantial loadings. Items with no bolded loadings had no loading greater than |.30|.

The SAD-P had a total omega of .96, indicating good internal consistency. The SAD-P was correlated with the Depression subscale in the CBCL (*r* =.65, *p* <.001), which suggests borderline acceptable (yet below the desired threshold) convergent validity. The SAD-P was also significantly positively correlated with the Conduct Problems subscale in the CBCL (*r* =.42, *p* < .001). Findings from the Fisher's Z transformation suggested that the SAD-P is more strongly related to the Depression subscale than the Conduct Problems subscale (*Z* = 2.12, *p* =.034).

## Discussion

The findings in the preliminary EFA examining all parent reports suggested that a one-factor model might be the most parsimonious and interpretable model. Although the overall fit indices did not indicate good fit according to traditional cut-offs, Montoya and Edwards (19) concluded that fit statistics should not be used solely to determine the number of factors in a EFA due to their tendency to favor a higher number of factors. Using fit change guidelines, interpretability, parsimony, loading patterns and correlated factors preliminarily supported the one-factor solution. While the one-factor model is the most pragmatic choice in this preliminary study, it is not a definitive statement about the construct of depression being one-factor.

We did not have the sample size to test measurement invariance, but we did qualitatively compare the one-factor model between parent reports for neurotypical and autistic youth. The one-factor solution for each group had slight differences in item loadings. Items assessing restlessness and aggression did not load substantially onto the one-factor in parent reports of neurotypical youth but did in the autism-only sample. While this difference is exploratory and may vary in subsequent analyses, it may support the idea that aggression is a more commonly endorsed depressive symptom in autistic people (7). Similarly, restlessness is generally more common in neurodivergent than neurotypical youth (21), which may explain stronger loadings in the autistic sample.

Some items that loaded well in the parent sample of neurotypical youth did not load well in the parent sample of autistic youth—specifically, feeling loved, guilty and motivated, and eating difficulties. Feeling loved and feeling guilty may not have loaded well in the autistic sample because of difficulty expressing emotions in autistic individuals (22). If autistic youth are not expressing feeling loved or feeling guilty, parents may have difficulty identifying and reporting on these emotions in their child. It is also possible that these four items did not load with the other items because they are measuring something other than depression. It should be noted that items which expressed observable behaviours loaded highly onto the factor. However, due to the exploratory, hypothesis generating nature of this study, these are preliminary findings.

Our findings highlighted some similarities with the one-factor model in the autistic and neurotypical parent reports. Research has shown that depressive symptoms such as irritability, sadness, and tearfulness (4) are common in neurotypical and neurodivergent populations. Similarly, symptoms of irritability, feeling like a failure, and overall negative affect and negative thoughts loaded highly in the SAD-P. This highlights the importance of including these symptoms of depression in assessments for both groups.

The SAD-P had excellent internal consistency. We found that the SAD-P had borderline acceptable (yet below the desired threshold) convergent validity with the Depression subscale of the CBCL. It was also moderately associated < .5 with the Conduct Problems subscale, which may be due to the overlap in symptoms of depression and symptoms of conduct disorders (22). A strong correlation of .8 or higher would be cause for concern, suggesting a lack of variability between the two measures. Thus, our preliminary analyses suggest good discriminant validity. The scores in the SAD-P were more strongly related to the Depressive Problems subscale than the Conduct Problems subscale. More research is needed to examine the validity of the SAD-P. Finally, since our preliminary validity analysis is based solely using parent report measures, validity using external criterion (e.g., structured interviews) is also needed.

Items examining a regression in previously attained skills did not load well in the SAD-P. This was true when examining all factor models of the SAD-P. It is possible that our sample was comprised of more low-support-needs autistic youth. We did not ask for the youths' level of support needs though we did ask parents to state the verbal ability of their child. Though verbal ability is not always consistent with functional ability or level of support needs (23, 24), the number of minimally-speaking participants was small (*n* = 9), which could explain why the preliminary EFA suggested poor loadings from these items. It is also possible our sample did not show regression because this is only present in clinically depressed individuals. Alternatively, contrary to popular belief, these symptoms may not represent depression in autistic individuals at all. A factor analysis on the SAD-P using reports of minimally-speaking autistic individuals should be conducted to examine whether symptoms of regression in previously attained skills load better in this sample.

### Limitations and future directions

It is important to note the low sample size when interpreting the results from the factor analysis. Generally, researchers recommend that a sample size ranges between *N* = 200 and *N* = 1000 (25, 26) or be 3 to 20 times the number of variables (26) when preforming an EFA. This may limit the findings of our EFA, and caution should be used when interpreting these preliminary results. Given our sample size we could not directly examine the measurement invariance of the SAD-P. This component is critical to scale development as it can provide crucial information into the scale's consistency across samples and allows for comparisons of depressive symptoms between groups (27). Additionally, we were unable to run multiple factor analyses to control for demographic variables such as age, gender, autistic traits, and co-occurring conditions. Future larger samples should test hierarchical or bifactor models (e.g., general depression and somatic/affective/cognitive subfactors), consider controlling for such variables when examining the SAD-P and should examine the measurement invariance of the SAD-P across diverse samples (e.g., sex, age group level of intellectual functioning/support needs, and presence of co-occurring psychiatric conditions). Though our sample size did not permit, future research should conduct a factor analysis solely on an autistic sample, using a neurotypical sample primarily as a comparison group. Also, due to the restricted nature of our preliminary analyses, we only removed items that loaded poorly in the first factor analysis. More extensive analyses need to be conducted to further reduce the poorly loading items.

We also did not confirm an autism diagnosis, although we did ensure that participants were diagnosed by medical professionals. We have noticed in online studies in autism there is a decreasing reliance on confirmation of diagnosis. Further research should determine if results remain consistent when a confirmation of diagnosis is present. We also did not collect or control for medical disorders that may have impacted depressive symptoms in this population.

Additional limitations include a potential selection bias and lack of demographic information. While we did recruit from across Canada and the U.S. in attempt to attain a diverse sample, it is possible that our online recruitment methods were more likely to reach a certain subset of the population. Most parents (94%) had at minimum completed a college degree, which is above the average educational level in Canada and the U.S. (28). Further, a failure to account for demographic information including racial identity and socioeconomic status limits our ability to comment on the generalizability of the findings with the SAD.

Ideally this study would have included both parent and youth reports, as parent's reports on their child's depression may differ from the child's themselves. Some studies have found that autistic youth's reports do significantly correlate with their parents' reports (24, 29), while others have found that parents report higher rates of depression in their youth than the youth themselves (6, 30). Future research should examine whether parent and child ratings are found to correlate with this measure.

Seven items demonstrated factor loadings below a |.30| and were therefore removed from the scale during the preliminary analyses. This resulted in 47 items retained for the neurotypical version and 48 items retained for the autistic version of the scale. As this study represents the first phase in the scale's development and validation, the primary objective at this stage was to examine the underlying factor structure and to remove any items that demonstrated weak or inadequate loadings. Given the large number of items remaining, there is a possibility of item redundancy, as well as an increased respondent burden associated with the length of the scale. This may limit its practicality in both research and clinical contexts. Accordingly, in subsequent phases of this validation, we aim to further reduce the total number of items by identifying and retaining a core set of items that demonstrate the strongest factor loadings and optimal discriminatory properties.

Since no broad internalizing measures (e.g., anxiety) were included in the validation of our scale, this current data supports a broad internalizing/depressive factor. Future research with the inclusion of additional scales (i.e., anxiety scales) and clinical interviews is needed to establish specificity to depression. Moving forward, an item analysis should be conducted to examine which items in the SAD-P predict the highest depressive symptoms. Given the low number of participants with confirmed depression, the preliminary measure should also be examined in autistic individuals with a confirmed diagnosis of depression. Confirmatory factor analyses should also be done to confirm the factor structure amongst larger and diverse samples. It would also be important to conduct a participatory action study, where autistic youth and their parents were consulted to comment on the validity of the items. Furthermore, parents should be asked about the comprehensibility of the items to ensure that they are understandable. With the use of the SAD-P, future research should continue examining the prevalence of co-occurring depression in autistic individuals, compare and explore symptom presentation to get a better understanding of how depression presents in autistic individuals, and evaluate treatment options for autistic individuals experiencing co-occurring depression.

## Data Availability

The raw data supporting the conclusions of this article will be made available by the authors, upon request and without undue reservation.

## Footnotes

^1^RMSEA, RMSR, and TLI were examined with caution, barring Montoya & Edward’s (20) recommendation in mind.
